# High-Quality Genome Assembly of *Olea europaea* subsp. *cuspidata* Provides Insights Into Its Resistance to Fungal Diseases in the Summer Rain Belt in East Asia

**DOI:** 10.3389/fpls.2022.879822

**Published:** 2022-05-17

**Authors:** Li Wang, Jianguo Zhang, Dan Peng, Yang Tian, Dandan Zhao, Wanning Ni, Jinhua Long, Jinhua Li, Yanfei Zeng, Zhiqiang Wu, Yiyun Tang, Zhaoshan Wang

**Affiliations:** ^1^Key Laboratory of Silviculture of the State Forestry Administration, Research Institute of Forestry, Chinese Academy of Forestry, Beijing, China; ^2^Collaborative Innovation Center of Sustainable Forestry in Southern China, Nanjing Forestry University, Nanjing, China; ^3^Kunpeng Institute of Modern Agriculture at Foshan, Foshan, China; ^4^Ecological Restoration and Industrial Development Workstation, Nujiang State Forestry and Grassland Bureau of Yunnan Province, Kunming, China

**Keywords:** genome assembly, *Olea europaea*, susceptibility gene, demographic history, nature selection

## Abstract

The olive tree (*Olea europaea* L.) is the most iconic fruit crop of the Mediterranean Basin. Since the plant was introduced to China in the 1960s, the summer rain climate makes it susceptible to pathogens, leading to some olive diseases. *Olea europaea* L. subsp. *cuspidata* is natively distributed in the Yunnan province of China. It has a smaller fruit size, lower oil content, and higher resistance compared to subsp. *europaea*, which makes subsp. *cuspidata* a critical germplasm resource to be investigated. Here, a high-quality genome of subsp. *cuspidata* with 1.38 Gb in size was assembled and anchored onto 23 pseudochromosomes with a mounting rate of 85.57%. It represents 96.6% completeness [benchmarking universal single-copy orthologs (BUSCO)] with a contig N50 of 14.72 Mb and a scaffold N50 of 52.68 Mb, which shows a significant improvement compared with other olive genomes assembled. The evaluation of the genome assembly showed that 92.31% of resequencing reads and an average of 96.52% of assembled transcripts could be aligned to the assembled genome. We found that a positively selected gene, *evm.model.Chr16.1133*, was shared with the results of transcriptome analysis. This gene belongs to the susceptible gene and negatively regulates the disease resistance process. Furthermore, we identified the *Cercospora* genus which causes the leaf spot disease in the infected leaves. The high-quality chromosome-level genomic information presented here may facilitate the conservation and utilization of germplasm resources of this subspecies and provide an essential genetic basis for further research into the differences in oil content and resistance between subsp. *cuspidata* and *europaea*.

## Introduction

The olive tree (*Olea europaea* L.) is the most iconic fruit crop of the Mediterranean Basin owing to its ecological, economical, and cultural significance. It constitutes a cornerstone of Mediterranean culture by its multiple past and present uses and omnipresence in traditional agrosystems (Gros-Balthazard et al., 2019). Virgin olive oil, the main product from olive trees and the principal component of the so-called Mediterranean diet, is recognized as a green health care cooking oil and is known as “liquid gold” for its high nutritional benefits, outstanding medical treatment and health care function, and exceptional organoleptic properties (Donaire et al., 2011). The olive plant was formally introduced into China in the 1960s and was mainly cultivated in subtropical areas (Han and He, 2007). In the Mediterranean region, the climate is hot and dry in summer and moderate and rainy in winter, and the sandy soil is neutral and alkaline. In China’s cultivation region, however, the climate is hot and rainy in summer, cold and dry in winter, and the soil is mostly acidic with a heavy texture (Wang et al., 2000).

Although more than 200 cultivars are now grown in China, most of them exhibit climate and soil incompatibility, accompanied by the emergence of some olive diseases caused by fungi and bacteria (Han and He, 2007), owing to the rainy and highly humid conditions which are conducive to the occurrence and development of diseases (Moral and Trapero, 2009). Some diseases are widespread in many olive plantations, such as *Cercospora cladosporioides* Sacc., *Cycloconium oleaginum Cast*, olive anthracnose, and leaf spot diseases. These diseases are considered to be important factors leading to the reduction of olive fruit yield and oil content. Leaf spot disease is prevalent in the Sichuan and Hubei provinces of China, where high rainfall from August to September leads to easy infection by pathogens that caused the withering and falling of leaves, resulting in decreased production and flowering in the next year. Besides, in some Mediterranean regions, it has also been found that the infection of olive by pathogenic fungi inflicts serious economic losses on olive-related industries (López-Escudero and Mercado-Blanco, 2011). These all indicate that improving olive resistance is important for the olive industry and is one of the most important aspects of olive breeding. Thus, finding a new germplasm resource with functional genes to adapt to the climate of East Asia to cultivate new olive varieties with resistance through hybridization and with the existing olive varieties is an important way to promote the development of the olive industry.

Up to now, three versions olive reference assembly have been released, including two olive cultivars of *Olea europaea* L. subsp. *europaea* vUar. *europaea* cv. ‘Farga’ (Cruz et al., 2016) and *Olea europaea* L. subsp. *europaea* cv. ‘*Arbequina*’ (Rao et al., 2021), and one oleaster of *Olea europaea* L. *sylvestris* (Unver et al., 2017), which generated genomes of 1.31 G, 1.30 G, and 1.48 G, with contig N50 values of 52.35 kb, 4.67 Mb, and 25.49 kb, respectively. Among these assembly versions, the contigs of “*Arbequina*” are completely anchored into 23 chromosomes by Hi-C which improved the olive genome assembly. All four samples belong to the Mediterranean climate zone. In fact, the olives are divided into six subspecies, including subsp. *europaea* (unique to the Mediterranean basin), subsp. *cuspidata*, subsp. *maroccana*, subsp. *laperrinei*, subsp. *cerasiformis* and subsp. *Guanchica* (Hannachi et al., 2009). Among them, subsp. *cuspidata* is known as native to a widespread area in southeast Europe and northeast Africa through southwest Asia to the Nujiang River Basin of Yunnan province in China (Green, 2002). Compared with subsp. *europaea*, it has a smaller fruit size and lower oil content but has better disease resistance and soil adaptability in the East Asian climate. Thus, subsp. *cuspidata* has been widely introduced to olive cultivation areas in China and is used as rootstock or a hybrid male parent to improve olive adaptability (Ye et al., 1981). Previous research shows that using subsp. *cuspidata* as rootstock grafting olive has not only increased survival rate and growth rate but also enhanced the adaptability of olive (Shi et al., 1991). In addition, an olive progeny issued from the cross *Olea europaea* L. subsp. *europaea* cv. “*Frantoio*” × subsp. *cuspidata*, is significantly superior to the parental species both in soil adaptability and disease resistance (Ma et al., 2014). Hybridization between the subspecies *europaea* and *cuspidata* has also been documented in other countries (Besnard et al., 2001). So far, resistance studies on olives mainly focus on the breeding of resistance varieties, only few related studies on identifying resistance genes have been reported.

The excellent resistance of subsp. *cuspidata* to pathogens may be ecologically owing to its long-term adaptation to the high temperature and highly humid environment in the Yunnan province of China; thus, it is a very promising germplasm for investigating resistance genes that can be used to enhance vitality and the ability of olive to resist the invasion of pathogens. Assembling the genome of this subspecies and comparing it with that of subsp. *europaea* will facilitate the conservation and utilization of germplasm resources of this subspecies, as well as further uncover the molecular basis of adaptive evolution and oil synthesis mechanisms and improve its marker-assisted breeding, etc.

In this study, we applied a combined strategy involving PacBio HiFi sequencing and Hi-C technologies to generate a chromosome-level assembly and then performed the population dynamics analysis, phylogenetic relationships, gene family expansion and contraction, whole-genome replication, unique genes analysis, positive selection, and transcriptome analysis. We found some positive selection genes were correlated with the term of response to stimulus, suggesting the relevant genes were under selection pressure after species differentiation that may be related to the environmental adaptation of subsp. *cuspidata*. We used specific genes to perform GO analysis and found some biological processes associated with oil synthesis. We also sampled the infected and healthy leaves of two cultivars to perform transcriptomic analysis and identified the *Cercospora* genus that may be causing leaf spot disease on the infected leaves of the two olive cultivars. The genes associated with resistance were identified in subsp. *cuspidata*, which can be an instance to investigate the genes against leaf spot disease between subsp. *europaea* and *cuspidata*, and is of great significance for improving the resistance of olives in the future. Given the significant differences between subsp. *cuspidata* and *europaea* in resistance and oil content, the chromosomal genome assembly constructed here is greatly conducive to the research of oil production and resistance mechanisms, which is instructive to the molecular breeding, phylogenetic, adaptability, and evolutionary biology research of olives.

## Materials and Methods

### Plant Materials

We sampled subsp. *cuspidata* individuals from the Yunnan province of China. A voucher specimen was deposited in the herbarium of the Forestry Research Institute of the Chinese Academy of Forestry. Young leaves were used for Illumina sequencing, PacBio HiFi sequencing, and the construction of Hi-C libraries. Four different tissues (stem, root, leaf, and fruit) were collected for RNA-seq analysis in order to assist genome assembly and annotation. In addition, we collected both the infected and healthy leaves of two cultivars (including “*Arbequina*” and “*Arbosana”*) in the olive plantation in the Hubei province of China. Three replicates of infected and healthy leaves were separately taken for each cultivar and were used for RNA extraction and transcriptome analysis. The construction of the Hi-C libraries was provided by Novogene Co., Ltd. while other sequencing services were provided by Berry Genomics Co., Ltd. (Beijing, China).

### Genome Sequencing and Transcriptome Sequencing

Short-insert-size (∼350 bp) libraries were constructed according to Illumina’s standard protocol and paired-end reads (2 × 150 bp) were sequenced using an Illumina HiSeq X Ten platform (Illumina Inc., San Diego, CA, United States). A 60 Kb DNA SMRTbell library was constructed and a circular consensus sequencing (CCS) was performed on the PacBio platform (HiFi) (Pacific Biosciences Inc., Menlo Park, CA, United States). Hi-C libraries (two-cell) were constructed with the restriction endonuclease DPNll and sequenced on the Illumina HiSeq X Ten platform.

PacBio HiFi long reads were used as a backbone scaffold in genome assembly using hifiasm (version 0.14-r312) that provides better assemblies than other available tools (Cheng et al., 2021). The Illumina short reads were used to investigate the genome characteristics (such as genome size and heterozygosity) before assembly and for assembly quality evaluation. The Hi-C reads were used to anchor the contig-level assembly into the final chromosome-level genome assembly (Burton et al., 2013). To obtain the uniquely mapped read pairs, the raw data were aligned with the assembled genome using BWA-MEM (version 0.7.17-r1188) (Li and Durbin, 2009). The valid Hi-C data were evaluated using HiC-Pro based on uniquely mapped read pairs (Servant et al., 2015).

All of the RNA-seq libraries were constructed using a VAHTS mRNA-seq v2 Library Prep Kit with an average insert fragment size of ∼350 bp, and sequenced on an Illumina Novaseq 6000 platform with a paired-end model.

### Quality Control of Sequencing Data

All sequencing data were filtered to eliminate low-quality bases and duplicated reads using different strategies based on the platforms used. For the Illumina Hi-Seq data, including genomic short-reads and RNA-seq reads, the PCR duplications of read pairs generated during the library construction process were first deleted. Then, adaptors were removed from the sequencing reads, and read pairs with more than 20% low-quality bases were deleted using Trimmomatic v0.33 (Bolger et al., 2014). If any read had more than 10% unknown bases, the read pair was excluded from further analysis (Chen et al., 2019). For Hi-C sequencing data, the same method used for Illumina Hi-Seq short-insert reads was adopted for filtering and then 3D was used for additional filtering. For PacBio HiFi long reads, subreads were directly filtered and corrected by the pbccs pipeline with default parameters^1^.

### Estimation of the Genome Size and Heterozygosity

Prior to the HiFi reads library-building sequencing, the investigation of the genome size and heterozygosity of subsp. *cuspidata* was carried out. The quality-filtered short fragments from the Illumina data were subjected to 21-mer frequency distribution analysis using Jellyfish v.2.2.10 (Marçais and Kingsford, 2011). We then performed genome analysis using GenomeScope2^2^ based on the results of Jellyfish. Ultimately, we obtained the genome information of subsp. *cuspidata* (Supplementary Figure 1), including genome size, heterozygosity, and repetitive sequence proportions.

### Genome Assembly

After filtering and correcting, the resulted HiFi CCS reads were subjected to hifiasm (v0.14-r312) for *de novo* assembly with default parameters^3^, and the redundant haplotigs were removed using Purge Haplotigs (Roach et al., 2018). The haploid contigs were scaffolded using the 3D *de novo* assembly (3D-DNA) software (Dudchenko et al., 2017). Briefly, the Hi-C reads were aligned to the draft genome assembly using Juicer; a 3D-DNA analysis was conducted to generate a candidate assembly; the candidate assembly was reviewed using Juicebox v1.9.8 Assembly Tools (JBAT) (Durand et al., 2016), and then corrected artificially on the basis of candidate assembly. Benchmarking Universal Single-Copy Orthologs (BUSCO) (v3.0.2) (Simão et al., 2015) program with eudicotyledons_odb10 database was used to assess the completeness of the genome and gene annotation. Furthermore, the filtered short reads generated from Illumina and the assembled transcripts were mapped against our assembly using BWA-MEM algorithm and HISAT2 (v2.1.0) (Kim et al., 2015), respectively.

### Repetitive Element Annotations

We employed the EDTA genome annotation pipeline (Ou et al., 2019) to annotate transposable elements (TEs) in the subsp. *cuspidata* genome, including retrotransposons and DNA transposons, in which long tandem repeats (LTRs) and long interspersed nuclear elements (LINEs) belonged to the former, while terminal inverted repeats (TIRs) and helitrons belonged to the latter, and were detected by RepeatModeler. A *de novo* repeat library was produced to identify repeat sequences using RepeatMasker (v4.0.7) (Tempel, 2012) and Repbase (Bao et al., 2015) according to the recommended parameter values.

### Gene Prediction and Functional Annotations

We mapped the RNA-seq data from the roots, stems, leaves, and fruits to the genome for predicting genes using the HISAT2 (v2.1.0) - StringTie (v1.3.5) pipeline and assembled the transcripts *de novo* by Trinity (Grabherr et al., 2011). Then, these transcripts were used to create transcript-based predictions with the PASA (v2.4.1) pipeline (Haas et al., 2003). The coding regions of the transcripts were annotated using a Transdecoder^4^. We also carried out homolog predictions. In such predictions, the protein sequences of *O. europaea* var. *sylvestris*, “*Arbequina*,” *Juglans regia*, *Sesamum indicum*, *Solanum tuberosum*, and *Vitis vinifera* species were mapped to the genome using Exonerate v2.2.0. GlimmerHMM (v3.0.4) (Majoros et al., 2004). SNAP (Johnson et al., 2008) and AUGUSTUS (v3.3.3) (Stanke et al., 2006) were trained with genes from the PASA results and used for *de novo* gene prediction. We merged the gene models from these sources using EVidenceModeler (v1.1.1) (Haas et al., 2008). To find functional clues for the protein-coding genes of subsp. *cuspidata*, the predicted protein sequences were compared with those in several public databases [GO, EuKaryotic Orthologous Groups (KOG), Kyoto Encyclopedia of Genes and Genomes (KEGG), SwissProt, Pfam databases, and Nr databases].

### Phylogenetic and Gene Family Analysis

Except for subsp. *cuspidata*, we chose one olive cultivar (“*Arbequina*”) and one oleaster (*O. europaea* var. *sylvestris*). In addition, we selected another 11 plant relative species, including *S. indicum*, *S. tuberosum*, *Eucalyptus grandis*, *Glycine max*, *Arabidopsis thaliana*, *Populus trichocarpa*, *Jatropha curcas*, *V. vinifera*, *Pistacia vera*, *Helianthus annuus*, and *Oryza sativa*, with *Oryza sativa* as outgroup. The protein sequences of all these species were downloaded from the NCBI. We first filtered these protein sequences with lengths of less than 100 bp to improve the alignment quality. OrthoFinder (v2.5.2) (Emms and Kelly, 2019) was then used to identify single-copy homologous genes and classify the protein sequences into families of 14 species with the key parameters “-M msa -S diamond -T raxml-ng,” where -M is the method for gene tree inference, -S is the alignment method, and -T is the tree inference method used. We inferred the phylogenetic relationship tree among 14 species and assessed the branch support with 100 bootstrap replicates using RAxML (Stamatakis, 2014). The divergence time was calculated using MCMCtree from the PAML package (Yang, 2007). In addition, the known divergence time between *P. trichocarpa* and *J. curcas* (77 Mya, CI:70–86 Mya) from the public resource TIMETREE^5^ was provided as calibration points in the analysis.

CAFE (v3.1) was used to analyze the expansion and contraction of the gene families (Han et al., 2013). We obtained the evolutionary tree and gene family clustering that were used to estimate the number of gene families of the ancestors in each phylogenetic tree branch, thereby predicting gene family contraction and expansion. The gene families with particularly large gene copy number variation were eliminated to decline parameter prediction errors using python script *cafetutorial_clade_and_size_filter.py*. The specific information of expansion and contraction gene families for the 14 species were finally obtained by applying the script *cafetutorial_report_analysis.py*, with these results used for later analyses. In addition, we uploaded the obtained gene family information to the OrthoVenn2 website for analysis^6^. Based on the gene families specific to subsp. *cuspidata* and ‘‘*Arbequina*’’ obtained from the above steps, we performed a functional enrichment analysis of GO terms using Fisher’s exact test^7^ to determine if any functional gene classes were overexpressed.

### Positive Selection Analysis

By comparing the protein sequences of subsp. *cuspidata* and “*Arbequina*,” we performed positive selection analysis using CODEML module in PAML, which can reveal the direction and strength of natural selection acting on the protein by estimating the non-synonymous and synonymous rates (*d*_*N*_ and *d*_*S*_) between two protein sequences and infer the positive selection of protein-coding genes. Prior to the CODEML program, the coding sequence of “*Arbequina*” with a length greater than 100 bp was first used to create a BLAST database using Makeblastdb, and then the protein sequence of subsp. *cuspidata* was used to align to the database for a screening of orthologous genes between the two species using Blastp with the e value of 1e-5. After obtaining the file with a.homolog suffix that included all of the co-orthologs, the name of the two-way optimal paired sequence was obtained with ParaAT, which is the input format of PAML. The synonymous and non-synonymous substitution rates and positive selection in sequences were estimated and detected using CODEML, and some of the variables within the control file were configured before the CODEML run. We set “icode = 0” to specify the universal genetic code, furthermore, we set “fix_omega = 0” and “fix_kappa = 0” to ensure that the parameters of the ω and the transition/transversion ratio were estimated separately *via* maximum likelihood. Since a comparison is made between the two subspecies, we only need to set the null model to find the gene with an omega (ω = *d*_*N*_/*d*_*S*_) value greater than 1, representing positive selection.

### Whole-Genome Duplication and Synteny Analysis

Oleaster, subsp. *cuspidate* and “*Arbequina*” were selected to perform whole-genome duplication (WGD) analysis by calculating fourfold synonymous (degenerative) third-codon transversion (4DTv) values and distributions of synonymous substitutions per synonymous site (Ks) within and between each species. The 4DTv rates of collinear gene pairs were calculated based on fourfold degenerate sites following the YN substitution model. *K*s values of the collinear orthologous gene pairs were calculated using KaKs_Calculator (v2.0) (Wang et al., 2010) with default parameters. The CIRCOS module of the TBtools (Chen et al., 2020) software was used to visualize the assembled chromosomes of the genome, gene density, GC content, repeat content, and gene synteny on individual pseudochromosomes. The nucmer (4.0.0beta2) program in MUMmer4 (Marais et al., 2018) was used to determine whether similar gene pairs were adjacent on the chromosome between subsp. *cuspidata* and “*Arbequina*,” ultimately obtaining all the genes in the synteny block.

### Demographic History Reconstruction

To estimate the population size history and split time of subsp. *cuspidata* and “*Arbequina*,” we utilized the resequencing date from one subsp. *cuspidata* and one “*Arbequina*” individual to perform SMC++ (Terhorst et al., 2016), which is capable of analyzing unphased genomes. The sequencing data of subsp. *cuspidata* were obtained from the genome survey analysis data in this study, and the sequencing data of “*Arbequina*” were downloaded from the Genome Warehouse in the National Genomics Data Center (NGDC) with the BioProject accession number PRJCA003222. We first estimated each population marginally using an estimate. Then, we created datasets containing the joint frequency spectrum for both populations. Finally, we refined the marginal estimates into an estimate of the joint demography using split. A generation time of 20 years (Diez et al., 2015) and a mutation rate of 7.77e-09 mutations per nucleotide per generation (Xie et al., 2016; Julca et al., 2020) were used to convert the scaled times and population sizes into real times and sizes.

### Identification of the Fungal Category

In order to identify the fungal species that caused the leaf spot of the two cultivars’ infected leaves, the unmapped reads of all infected leaves in “*Arbequina*” and “*Arbosana*” were extracted to perform *de novo* genome assembly. The clean Fastq data of infected leaves were first mapped to the assembled genome and olive chloroplast and mitochondrial sequences with HISAT2. The unmapped reads were then extracted using samtools with the key parameters “-b -h -f 4,” and performed *de novo* assembly using Trinity (v2.1.1). After this, we downloaded the Nr database from NCBI and extracted the fungi subset using TaxonKit with the parameter of “-j 8 –ids 4751,” in which, “–ids 4751” represents the subset of fungi. The subset was used to create a BLAST database using Makeblastdb, and then the assembled sequences were aligned to the fungi database using Blastp with the e value of 1e-5.

### Differential Gene Expression Analysis

“*Arbequina*” and “*Arbosana*” are the most widely cultivated in plantations due to their high production (Centeno et al., 2019). We thus collected the infected and healthy leaves from these two cultivars in September for differential gene expression analysis, because olives were susceptible at this time. The transcriptome clean Fastq data from infected and healthy leaves were mapped to the assembled genome with HISAT2. The alignments were used for transcript assembly using StringTie, which assembles the genes for each data set separately and estimates the expression levels of each gene and isoform. All the gene structures found in any of the samples were merged together with the key parameter of “stringtie -merge,” and then, all the transcripts and abundances were obtained using Ballgown (Pertea et al., 2016). The result of transcript quantification obtained from Ballgown was converted to the count matrices of genes and transcripts with the command of “python2 prepDE.py -i ballgown,” in which the script *prepDE.py* was downloaded from http://ccb.jhu.edu/software/stringtie/dl/prepDE.py. Finally, differential gene analysis was performed with the count data using DEseq2 package in R, which provides methods to test for differential expression by using negative binomial generalized linear models (Love et al., 2014). We separately grouped these data into two groups of healthy and infected leaves for each cultivar and screened differentially expressed genes (DEGs) using DESeq2 with an adjusted *p*-value < 0.05 and the absolute value of a log2(FC) > 1 (Love et al., 2014), which were also used for GO analysis. Differential expression genes were further classified as upregulated and downregulated based on their log fold change (FC) values. Genes with an FC value greater than zero were considered upregulated, while those with less than zero were thought to be downregulated. Further, we calculated the FPKM (Fragments per Kilobase Million) values using Ballgown to validate the expression of each gene in infected and healthy leaves of two cultivars. Genes were considered low expressed if they had an FPKM value between.125 and 1, medium expressed if they had a value between 1 and 10, and highly expressed if the value was above 10 (Hackett et al., 2012). We also sampled three replicates of healthy leaves of subsp. *cuspidata* to compute the FPKM values to understand the expression of differential genes in subsp. *cuspidata*.

## Results

### *De novo* Assembly of the subsp. *cuspidata* Genome

**FIGURE 1:**
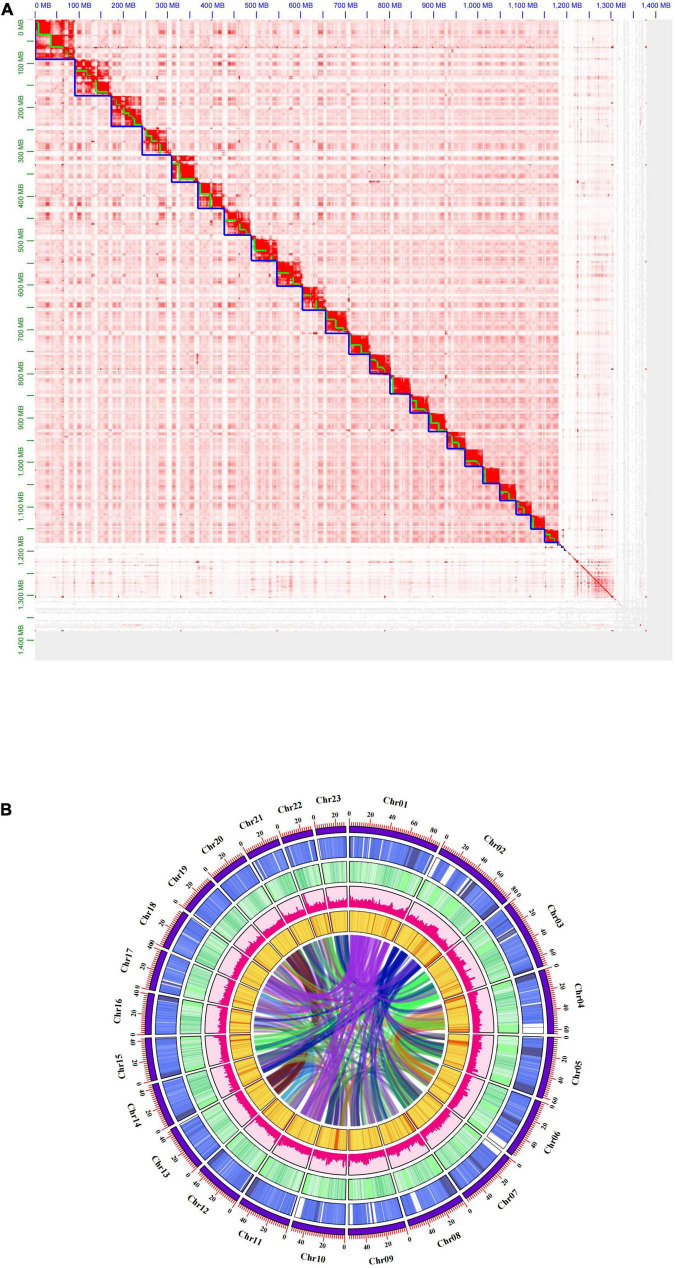
Genome-wide Hi-C interaction heatmap and Genomic landscape. **(A)** Hi-C interaction heat map between 23 chromosomes for the subsp. *cuspidata* genome. **(B)** Genomic landscape of subsp. *cuspidata* chromosomes. Visualize the genome assembly chromosome, gene density, GC content, repeat content, SNP density, and gene collinearity on a single pseudochromosome from the outer ring to the inside.

We obtained ∼253.5 Gb clean Fastq data for the Illumina short reads. To resolve any difficulties that may arise during the genome assembly process, the Kmer-based method was used to perform genome survey analysis to estimate the genome size and heterozygosity of the subsp. *cuspidata* genome using Illumina short reads. We counted the number of each 21-mer with Jellyfish, and the frequency distribution was plotted in Supplementary Figure 1. The subsp. *cuspidata* genome size was then estimated to be 1.18 Gb with 0.36% heterozygosity, and the coverage is ∼34.7-fold relative to the actual assembly results. To obtain a high-quality genome assembly, a total of ∼44.72 Gb of PacBio HiFi long reads (reads: 3,294,182, average N50: ∼14.85 Kb) were generated and subjected to hifiasm for *de novo* genome assembly. After assembly and deduplication, the consensus sequences resulted in a contig level assembly of 1.38 Gb spanning 3,073 contigs, with a contig N50 of 14.7 Mb and the longest contig of 38.04 Mb (Table 1). We obtained ∼450 Gb of Hi-C Fastq clean data with the effect rate of 34.61%, and used it for chromosome construction using 3D *de novo* assembly. A total of 1.18 Gb sequences spanning 2,695 scaffolds were finally anchored onto 23 pseudochromosomes (Figure 1), with a scaffold N50 of 52.68 Mb and the longest scaffold of 90.13 Mb (Table 1). The mounting rate was 85.57% (Table 2), and the average GC content was 0.36. The BUSCO results showed that more than 2,048 (96.6%) genes were completely recalled, of which 81% were single-copy and 15.6% originated from duplication (Table 3). A total of 879,715 transcripts were acquired, with an average of 96.52% reads located in the assembled genome (Supplementary Table 1). The mapping rate of resequencing reads exceeded 92.31% of the whole genome.

**TABLE 1:** Statistics of assembled subsp. *cuspidata* genome.

Term	Contig size (bp)	Contig number	Scaffold size (bp)	Scaffold number
N90	350,652	257	885,961	52
N80	2,313,398	94	34,033,801	21
N70	6,370,930	55	40,664,807	17
N60	11,521,701	40	44,152,604	14
N50	14,716,965	30	52,676,021	11
Max length (bp)	38,043,138		90,127,509	
Total size (bp)	1,379,115,243		1,379,304,243	
Total number	3,073		2,695	
Average length	448,784.65		511,801.20	

**TABLE 2:** Statistics of chromosomal level assembly of subsp. *cuspidata.*

Chr ID	Length (bp)	Chr ID	Length (bp)	Chr ID	Length (bp)
Chr1	90,127,509	Chr9	57,282,915	Chr17	40,664,807
Chr2	83,097,257	Chr10	52,971,700	Chr18	39,899,167
Chr3	70,287,963	Chr11	52,676,021	Chr19	37,263,953
Chr4	64,129,678	Chr12	47,592,757	Chr20	37,211,276
Chr5	61,350,988	Chr13	45,546,967	Chr21	34,033,801
Chr6	59,983,315	Chr14	44,152,604	Chr22	31,166,573
Chr7	58,685,853	Chr15	42,848,148	Chr23	29,903,841
Chr8	58,506,042	Chr16	40,951,526		
Total chromosome level contig length			1,180,334,661		
Total contig length			1,379,304,243		
Chromosome length/Total length			85.57%		

**TABLE 3:** Completeness assessment of subsp. *cuspidata* genome by BUSCO.

Library	eudicotyledons_odb10
Complete BUSCOs (C)	2048
Complete and single-copy BUSCOs (S)	1717
Complete and duplicated BUSCOs (D)	331
Fragmented BUSCOs (F)	24
N50Missing BUSCOs (M)	49
Total BUSCO groups searched	2121
Summary (Complete BUSCOs/Total BUSCOs)	96.6%

### Repetitive Sequences, Gene Prediction, and Functional Annotations

We annotated all repetitive sequences to further characterize the genome of subsp. *cuspidata* by integrating *de novo* and homology-based approaches. We predicted 69.61% of the genome as transposable elements. DNA transposons were the most abundant characterized elements, in which, TIRs accounted for 30.2% and non-TIRs accounted for 3.6%. In retrotransposons, LTRs accounted for 29.5% and non-LTRs accounted for 0.17% (Table 4).

**TABLE 4:** Statistics of TE annotated repeat sequences in subsp. *cuspidata* genome.

Class	Sub-Class	Type	Length (bp)	Percent (%)
**Retrotransposons**	**LTR**	Ty1/Copia	137,408,274	9.96%
		Ty3/Gypsy	205,089,955	14.87%
		unknown	63,995,280	4.64%
	**Non-LTR**	LINE	1,905,667	0.14%
		unknown	423,876	0.03%
**DNA transposons**	**TIR**	CACTA	18,631,143	1.35%
		Mutator	347,273,079	25.18%
		PIF/Harbinger	19,703,347	1.43%
		Tc1/Mariner	2,351,832	0.17%
		hAT	28,621,867	2.08%
	**Non-TIR**	helitron	48,941,818	3.55%
	**Total**		960,043,533	69.61%

A total of 46,904 protein-coding genes were predicted in the current assembly, and then we implemented the gene function annotation using GO, KEGG, KOG, SwissProt, Pfam annotation, and Nr annotation databases. From this analysis, most of the predicted genes were functionally annotated in these databases (Table 5).

**TABLE 5:** Statistics of functional annotation of protein-coding genes in subsp. *cuspidata* genome.

Database	Annotated gene number	Percent (%)
GO	26,012	57.60
KEGG	8,327	18.44
KOG	8,941	19.80
SwissProt	33,018	73.12
Pfam annotation	32,739	72.50
Nr annotation	45,146	99.98

### Genome Evolution, Phylogeny, and Synteny Analysis

**FIGURE 2:**
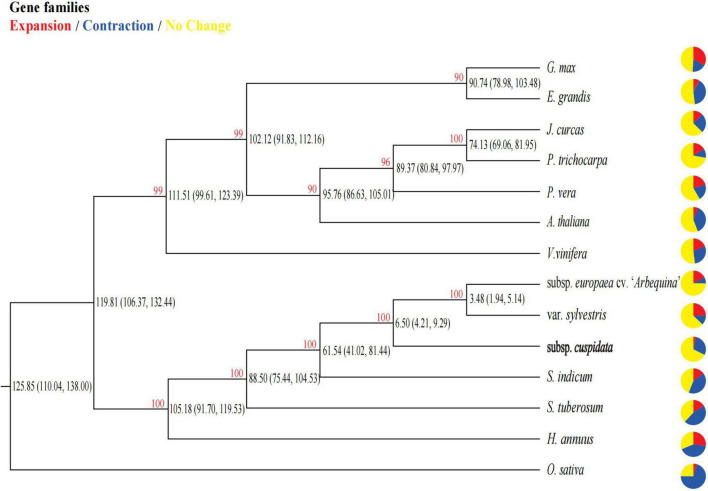
Phylogenetic relationship and divergence time among species. Pie charts show the proportion of gene families that are expanded (red), contracted (blue), and conserved (yellow) across all gene families in the 14 species. The red number in each node represents the bootstrap value. The number in parentheses in each internal node indicates the estimated divergence time interval (in millions of years).

A total of 65,396 gene families were obtained in all species, namely, subsp. *cuspidata*, “*Arbequina*,” var. *sylvestris*, *S. indicum*, *S. tuberosum*, *E. grandis*, *G. max*, *A. thaliana*, *P. trichocarpa*, *J. curcas*, *V. vinifera*, *P. vera*, *H. annuus*, *O. sativa*. We reconstructed a phylogenetic tree based on a concatenated sequence alignment of all single-copy genes which are shared by these species and estimated their divergence time. All the relationships were well supported with > 90% bootstrap values (Figure 2). As expected, oleaster and “*Arbequina*” were grouped together, and the splice time between them occurred approximately 3.48 (1.94, 5.14) million years ago (Mya), subsp. *cuspidata* diverged from them about 6.5 (4.21, 9.29) Mya, while olive diverged from *S. indicum* about 61.54 (41.02, 81.44) Mya.

**FIGURE 3:**
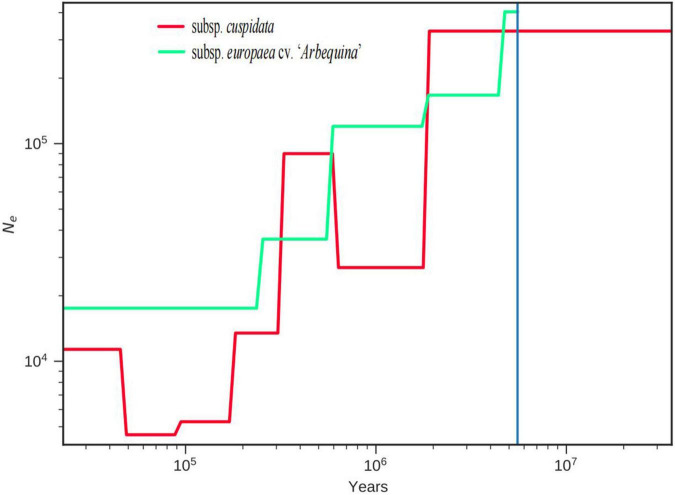
Population history analysis of subsp. *cuspidata* and “*Arbequina*”. SMC++ estimates the effective population size (*Ne*) changes for subsp. *cuspidata* and *“Arbequina*,” and estimates the splice time between subsp. *cuspidata* and *Arbequina*”.

The population demographic history inferred with SMC++ software showed evidence for a considerable and continuous decline in both population sizes. The population of subsp. *cuspidata* started approximately 13 Mya (Figure 3), closing to the high central plateau of the Qinghai-Tibet Plateau timeframe (∼10–13 Mya) (Zhang et al., 2010). The splice time between subsp. *cuspidata* and “*Arbequina*” was approximately 5.5 Mya, which was generally consistent with the timing of the phylogenetic tree.

**FIGURE 4:**
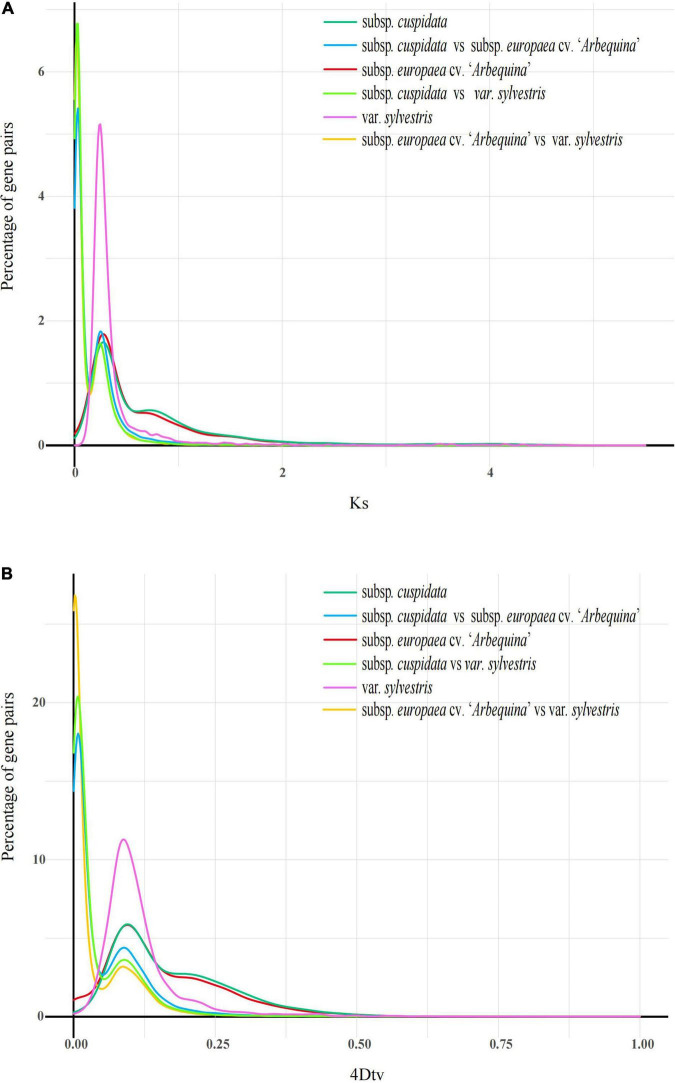
Whole-genome duplication (WGD) analysis. **(A)** *K*s distributions analysis. Peaks of intraspecies *K*s distributions indicate whole genome polyploidization events, and peaks of interspecies *K*s distributions indicate speciation events. **(B)** The 4DTv distribution of gene pairs in subsp. *cuspidata* and other genomes. The *x*-coordinate is the 4DTv value, and the *y*-coordinate represents the proportion of genes corresponding to the 4DTv values.

Whole-genome duplication (WGD) is seen as an important factor with a significant effect on plant genome evolution (Mcgrath and Lynch, 2012). To further understand the genomic evolution of subsp. *cuspidata*, “*Arbequina*” and oleaster, we performed WGD analysis; the collinearity of inter- and intra- olive genomes provided evidence of these three species’ WGD events (Figure 4). By determining the distribution of 4DTv and *K*s values, we detected one main peak within subsp. *cuspidata* (the peak of 4DTV: ∼0.092, *K*s: ∼0.389), “*Arbequina*” (4Dtv: 0.091, *K*s: ∼0.271), and oleaster (4Dtv: ∼0.085, *K*s: ∼0.221), indicating that all three species had experienced one WGD event, which was similar to the result of previous research (Rao et al., 2021). Following that, species divergence occurred. The divergence of subsp. *cuspidata* - oleaster occurred at a peak of *K*s ∼0.137, followed by subsp. *cuspidata* – “*Arbequina*” (*K*s, ∼0.135) and “*Arbequina*” - oleaster (*K*s, ∼0.013) divergence.

**FIGURE 5:**
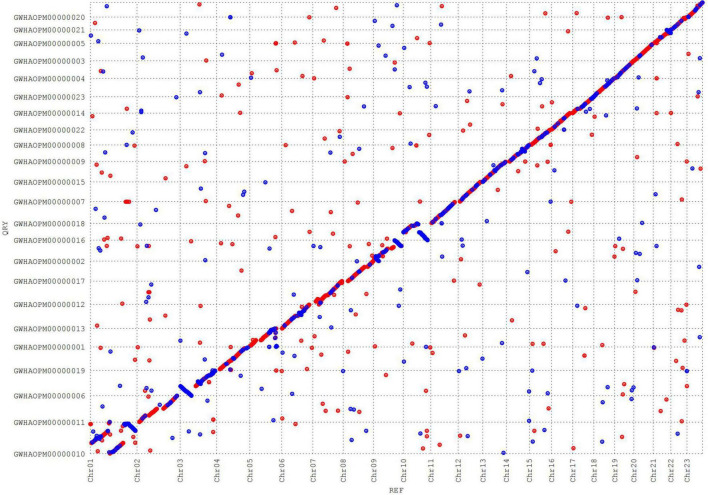
Dot synteny diagram of chromosomes in subsp. *cuspidata* and “*Arbequina*”.

Synteny analysis revealed a high linear relationship between subsp. *cuspidata* and “*Arbequina*.” A total of 43,711 genes in subsp. *cuspidata* were found to have synteny with “*Arbequina*.” The synteny between chromosomes was partially dislocated (Figure 5), which may have been caused by two reasons: First, the “*Arbequina*” adopted the sequencing technology of Oxford Nanopore, whose error rate was as high as ∼40%, much higher than PacBio HIFI (lower than 1%) (Laver et al., 2015; Ye and Ma, 2016). Second, the genome of “*Arbequina*” was assembled by merging the results of the three different software (including Canu, Wtdgb, and SMARTdenovo), which may have introduced further errors.

### Comparative Genomics Analysis

**FIGURE 6:**
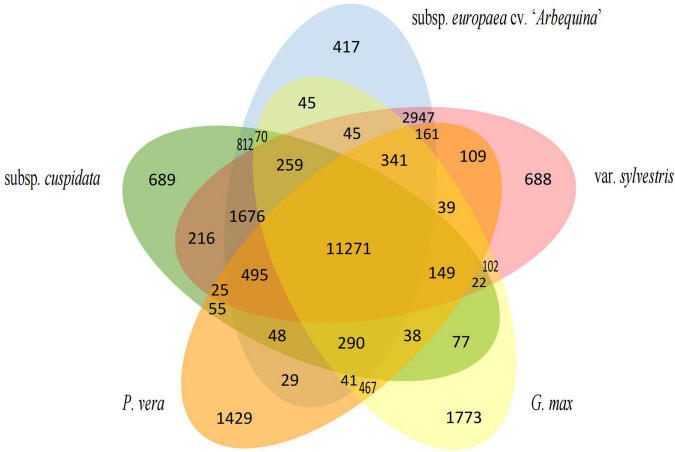
Petal diagram of the gene families for six oil species. The middle number represents the gene families shared by all species and the number of gene families unique to each species is on the side.

We compared six oil species that aimed to search for genes associated with oil production. A total of 10,813 gene families were shared by these six species, and 681 gene families were unique in subsp. *cuspidata*, 394 in “*Arbequina*,” 656 in *O. europaea* var. *sylvestris*, 477 in *S. indicum*, 2,853 in *H. annuus*, and 1,932 in *G. max* (Figure 6). These specific gene families of subsp. *cuspidata* and “*Arbequina*” were then separately annotated to GO terms. In “*Arbequina*,” unique genes were grouped into annotations of nine biological processes, 8 cellular components, and nine molecular functions (Supplementary Figure 2). In the biological process group, we obtained 384 biological process descriptions, of which 51 were significantly expressed (*P* < 0.05) containing 81 genes (Supplementary Table 2). Interestingly, we found some significant expression processes associated with lipid biosynthetic, including the metabolic and/or catabolic process of *S*-glycoside and glycosinolate and the biosynthetic and metabolic process of the acetyl-CoA. Lipid is one of the major carbon storage compounds (Li et al., 2010), while glycogen is one of the major metabolites for carbon storage in many plants (Govindprasad et al., 2017). The acetyl-CoA is the most abundant short-chain acylCoA in olive fruit (Sanchez-Ortiz et al., 2012) and serves as a precursor for fatty acid synthesis (Salas et al., 2013; Priore et al., 2014). Thus, both glycogen and acetyl-CoA play an important role in fatty acid synthesis. This result suggests the important position of genes associated with oil synthesis in genes specific to “*Arbequin*.” Similarly, in subsp. *cuspidata*, unique genes were grouped into annotations of 9 biological processes, 8 cellular components, and 10 molecular functions (Supplementary Figure 3). In the biological process group, 389 biological process descriptions were obtained, of which 132 were significant and contained 1,415 genes. We also found some significant expression of the progress related to lipid synthesis, such as the biosynthetic and/or metabolic process of glycosyl compound, carbohydrate derivative, aromatic compound, organic cyclic compound, cellular lipid, and trehalose (Supplementary Table 3).

We conducted a positive selection analysis between “*Arbequina*” and subsp. *cuspidata*. A total of 38,158 single copy orthologous genes were compared and 2,777 genes accounting for 7.28% were finally identified under positive selection (*d*_*N*_/*d*_*S*_ > 1) in subsp. *cuspidata*. GO enrichment analyses show that these genes were categorized into 37 functional groups, including 17 biological processes, 9 cellular components, and 11 molecular function annotations (Supplementary Figure 4). Significantly, we found a term of response to stimulus (GO: 0050896) with 66 genes in biological process, such as response to water, response to inorganic substance, response to endogenous stimulus, response to biotic stimulus, defense response (Supplementary Table 4), suggesting the relevant genes were under selection pressure after species differentiation that may be related to the environmental adaptation of subsp. *cuspidata*.

### Identification of the Fungal Genus

**FIGURE 7:**
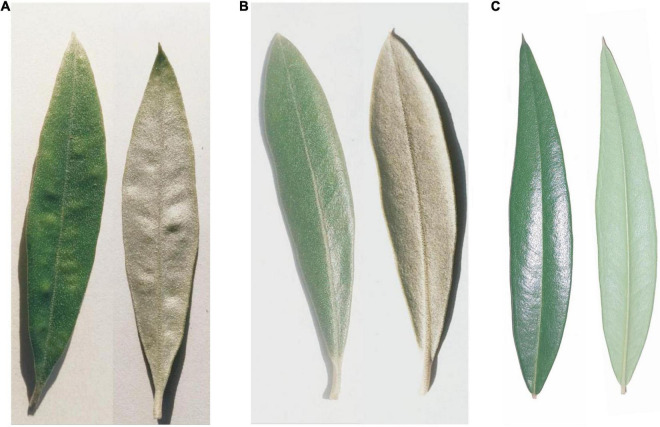
The leaves of the three subspecies. The symptom of infected leaves is identical in “*Arbequina*” and “*Arbosana*”. **(A,B)** Represents the front and back of the infected and healthy leaves, respectively. **(C)** Represents the front and back of the healthy leaves of subsp. *cuspidata*.

Compared with healthy leaves, the symptom of the infected leaves is pathogen-induced spot (Figure 7). To identify the fungal species, we extracted the unmapped sequences from all infected leaves for *de novo* genome assembly and aligned them to the constructed fungal library. We found the fungi in genus *Cercospora* presented in all six alignment results and with the highest identity, including *Cercospora beticola*, *Cercospora zeina*, and *Cercospora kikuchii*, they were well supported with > 40% identity (Supplementary Tables 5, 6). As we expected, three fungi were causing foliar diseases. *Cercospora* is known to be one of the main groups of plant pathogenic fungi, which can cause necrotic leaf spots in many plants (Groenewald et al., 2013). Since the symptom of leaf spot was also appeared in the infected leaves we collected, this result is largely reliable.

### Differential Gene Analysis of Transcriptome

We performed differential gene analysis for the infected and healthy leaves of the two cultivars and obtained 248 and 475 DEGs. Among these, 117 and 172 genes were upregulated and 131 and 303 genes were downregulated in “*Arbequina*” and “*Arbosana*,” respectively (Supplementary Tables 7, 8). Moreover, 49 common genes were differentially expressed in two cultivars. To gain further insight into the function of the 49 genes in subsp. *cuspidata*, we performed GO enrichment analysis, categorizing the 49 DEGs into 19 functional groups, which included seven biological processes, seven cellular components, and five molecular function annotations (Supplementary Figure 5). Among them, we found only one gene (*evm.model.Chr16.1133*) with a term of response to stimulus in the biological process group; significantly, this gene also underwent positive selection. This gene sequence was then aligned to *A. thaliana* using Blastp with the e value of 1e-5, indicating *evm.model.Chr16.1133* is homologous to *AtMLO6* (AT1G61560), with a 61.78% identity. In our results, *evm.model.Chr16.1133* gene was moderately expressed in the healthy leaves of “*Arbequina*” and “*Arbosana*” (the mean FPKM value was 1.793 and 3.150 of three duplicates, respectively), but had a low expression in infected leaves of the two cultivars (the mean FPKM value was 0.558 and 0.818, respectively) (Table 6), and the log2(FC) value was separately –1.702 and –1.917 (Supplementary Tables 7, 8), indicating the negative regulatory role of it against pathogens, which was in agreement with previous studies (Bai et al., 2008; Delventhal et al., 2011). Furthermore, this gene had also a low expression in subsp. *cuspidata* healthy leaves (mean FPKM: 0.583), implying that the low expression of this gene may be related to good resistance in subsp. *cuspidata*.

**TABLE 6:** Statistics of the FPKM values for *evm.model.Chr16.1133*.

Species	Healthy leaves	Mean	Infected leaves	Mean
	1.948		1.047	
‘*Arbequina*’	0.999	1.793	0.240	0.558
	2.433		0.388	
	2.871		1.212	
‘*Arbosana*’	2.239	3.150	0.677	0.818
	4.341		0.566	
	0.449		-	
subsp. *cuspidata*	0.870	0.583	-	-
	0.431		-	

According to an underway olive-related study, the sequences of *evm.model.Chr16.1133* gene were separately obtained from 29 subsp. *cuspidata* and 25 olive cultivar individuals. We computed polymorphic sites, the values of Tajima’s *D* and nucleotide polymorphism (θ_π_) using DnaSP. We found no polymorphic sites of this gene in subsp. *cuspidata*, while the cultivars showed a higher polymorphism with the Tajima’s *D* value of 0.929 and θ_π_ value of 0.003 (Table 7), indicating that severe natural selection led to no polymorphism of this gene in subsp. *cuspidata*.

**TABLE 7:** Genetic diversity of *evm.model.Chr16.1133* in 29 *cuspidata* and 25 cultivar individuals.

Species	Tajima’s *D*	θ_π_	Polymorphic sites
subsp. *cuspidata*	–	–	–
Cultivars	0.929	0.003	21

## Discussion

### Phylogenetic Analysis

Olive is a world-renown tree species owing to its economic, ecological, cultural, and scientific values. The phylogenetic analysis showed that the ancestor of oleaster and “*Arbequina*” was a sister of subsp. *cuspidata*, and the divergence between them was approximately 6.5 (4.21, 9.29) Mya. SMC++ results showed the split time of 5.5 Mya between “*Arbequina*” and subsp. *cuspidata* and was similar to the phylogenetic analysis, which also showed a considerable decline in both population sizes, and the subsp. *cuspidata* population started approximately 13 Mya. These results were close to the formation of the high central plateau of the Qinghai Tibet Plateau (QTP) at 10–13 Mya. In the late Cretaceous period of approximately 60 Ma BP, however, most of the QTP was still in the ancient Mediterranean at that time. It had a hot tropical-subtropical climate and was a region where thermophilic plants developed and flourished at that time (Sun and Li, 2003), where *Canarium* was one of the common floras (Mai, 1989; Zheng, 1989). The retreat of the ancient Mediterranean and the uplift of the QTP changed the Asian climate system and promoted the formation of inland drought in Asia (Peng, 2013). Since subsp. *cuspidata* may be the remaining species of paleo-Mediterranean flora that originated from the ancient Mediterranean region, we thus speculated that the uplift of the QTP may have caused the differentiation between them, and potentially reduced subsp. *cuspidata* historic population sizes.

### Determination of the Fungal Genus

The rainy summer climate in East Asia is conducive to the reproduction of pathogens, and the introduced olives are thus susceptible to pathogen invasion, leading to a decline in fruit production and even the trees’ death. The fungi causing the leaf spot disease in the two olive cultivars were identified to be the *Cercospora* genus, which was known as one of the main groups of plant pathogenic fungi. *C. beticola* is a worldwide distributed fungal disease and severely destroys the leaves of *Beta vulgaris* L., causing leaf spots and further resulting in the reduction of production and sugar content (Shane, 1992). *C. zeina* is distributed in many countries, it causes gray leaf spot of maize and leads to the reduction of maize yield (Meisel et al., 2009). *C. kikuchii* occurs in all soybean producing regions around the world, it causes purple seed stain on seed pods and seeds, and leaf blight on leaves and petioles, which has seriously affected the quality of soybean (Takeshi and Tomohiro, 2021). These three fungi are associated with foliar diseases, which is consistent with the symptom of the infected leaves that we collected. We thus speculate that the *Cercospora* genus may be causing the leaf spot disease in the infected leaves of the two olive cultivars.

### Identification of the Susceptibility Gene

Compared with olive cultivars, subsp. *cuspidata* has lower oil content but higher resistance to fungal diseases and abiotic stress (Hannachi et al., 2009; Trapero et al., 2015). Thus, it is a valuable genetic resource to investigate the differences in oil content and resistance between subsp. *europaea* and *cuspidata*. Here, we performed a GO analysis for the positive selection of genes and found 66 genes belonging to the term of response to stimulus, indicating that the genes associated with environmental adaptation were under selection pressure in subsp. *cuspidata.* Interestingly, one of the positive selected genes, *evm.model.Chr16.1133*, belongs to the term of defense response (GO:0006952); it is also found in the results of transcriptome differential gene analysis. *AtMLO6* is the homolog of this gene in *A. thaliana*; it is a well-characterized susceptibility gene belonging to the mildew resistance locus O (MLO) gene family, which is a class of single-gene controlled recessive disease resistance genes that negatively regulates the disease resistance process and leaf cell death in plants (Buschges et al., 1997; Piffanelli et al., 2002). The *MLO* gene was originally found in barley and also found in some plants, such as *A. thaliana* (Vogel et al., 2006), *Rosa multiflora* (Xiang et al., 2018), *Pisum sativum* (Humphry et al., 2011), *Malus domestica* (Pessina et al., 2014), and *V. vinifera* (Feechan et al., 2008). The loss-of-function mutants, *mlo*, were obtained by using X-ray, which has a broad-spectrum resistance to powdery mildew (*Blumeria graminis* f.sp. *hordei*) (Freisleben and Lein, 1942). Moreover, the downregulation of the *MLO* gene also caused a higher resistance to powdery mildew in barley (Delventhal et al., 2011). In addition, silencing *SlMLO1* gene confers robust powdery mildew resistance in tomato (Bai et al., 2008). All these indicate the important role of the *MLO* gene in plant disease resistance. Consistent with previous studies, this gene’s expression in infected leaves was lower than that in the healthy leaves of the two olive cultivars, suggesting this gene’s negative regulatory role. It is worth mentioning that subsp. *cuspidata* has a lower expression of this gene than the two cultivars in healthy leaves. Besides, we computed polymorphic sites, Tajima’s *D* and θ_π_ for this gene sequences of all 29 subsp. *cuspidata* and 25 olive cultivar individuals. No polymorphism site was found in subsp. *cuspidata*. All results indicate that this gene has undergone strict positive selection and provide a validated explanation for the higher resistance against pathogens in subsp. *cuspidata*.

Overall, we used high-accuracy PacBio HiFi sequencing and Hi-C technologies to assemble a chromosome-level genome of subsp. *cuspidata*, which significantly improved the assembly quality of olive. We performed transcriptome analysis and identified the fungi genus of infected leaves as well as a susceptible gene that was also found in our positive selection analysis. Given the characteristics of smaller fruit size and lower oil content but higher resistance of subsp. *cuspidata* compared with those of subsp. *europaea*, the genome assembly presented here will provide a valuable molecular resource to investigate the differences of oil content and resistance between them.

## Data Availability Statement

The datasets presented in this study can be found in online repositories. The names of the repository/repositories and accession number(s) can be found below: http://db.cngb.org/cnsa/, CNP0002655.

## Author Contributions

ZWa and JZ planned and designed the research. LW, JZ, DP, YTi, DZ, WN, JLo, JLi, and YZ analyzed the data. LW and ZWa contributed to writing the manuscript. All authors contributed to the article and approved the submitted version.

## Conflict of Interest

The authors declare that the research was conducted in the absence of any commercial or financial relationships that could be construed as a potential conflict of interest.

## Publisher’s Note

All claims expressed in this article are solely those of the authors and do not necessarily represent those of their affiliated organizations, or those of the publisher, the editors and the reviewers. Any product that may be evaluated in this article, or claim that may be made by its manufacturer, is not guaranteed or endorsed by the publisher.

## Footnotes

1
https://github.com/PacificBiosciences/ccs

2
http://qb.cshl.edu/genomescope/

3
https://github.com/chhylp123/hifiasm

4
https://github.com/TransDecoder/TransDecoder

5
http://www.timetree.org

6
https://orthovenn2.bioinfotoolkits.net/cluster-venn

7
https://www.omicshare.com/tools/Home/Soft/gogsea

## Supplementary Material

Supplementary Figure 1 Graph of the k-mer distribution (*K* = 21) generated using GenomeScope2.0. The big peak at the coverage of ∼60 in the graph is the homozygous portion of the genome, which accounts for the strands of the DNA having identical 21-mers. The smaller shoulder to the left of the peak corresponds to the heterozygous portion of the genome, which accounts for the strands of the DNA having different 21-mers. If the genome is highly heterozygous, the height of the shoulder peak would be closer to that of the homozygous peak.

Supplementary Figure 2 GO terms (level 2) distribution of “*Arbequina*” unique genes.

Supplementary Figure 3 GO terms (level 2) distribution of subsp. *cuspidata* unique genes.

Supplementary Figure 4 GO terms (level 2) distribution of subsp. *cuspidata* positive selection genes.

Supplementary Figure 5 GO terms (level 2) distribution of the 49 shared DEGs between “*Arbequina*” and “*Arbosana*”.
