# Supplementary figures and images for: High-Quality Genome Assembly of Olea europaea subsp. cuspidata Provides Insights Into Its Resistance to Fungal Diseases in the Summer Rain Belt in East Asia
Source: Front Plant Sci. 2022 May 17;13:879822. doi: 10.3389/fpls.2022.879822 (PMC9152427; doi:10.3389/fpls.2022.879822)

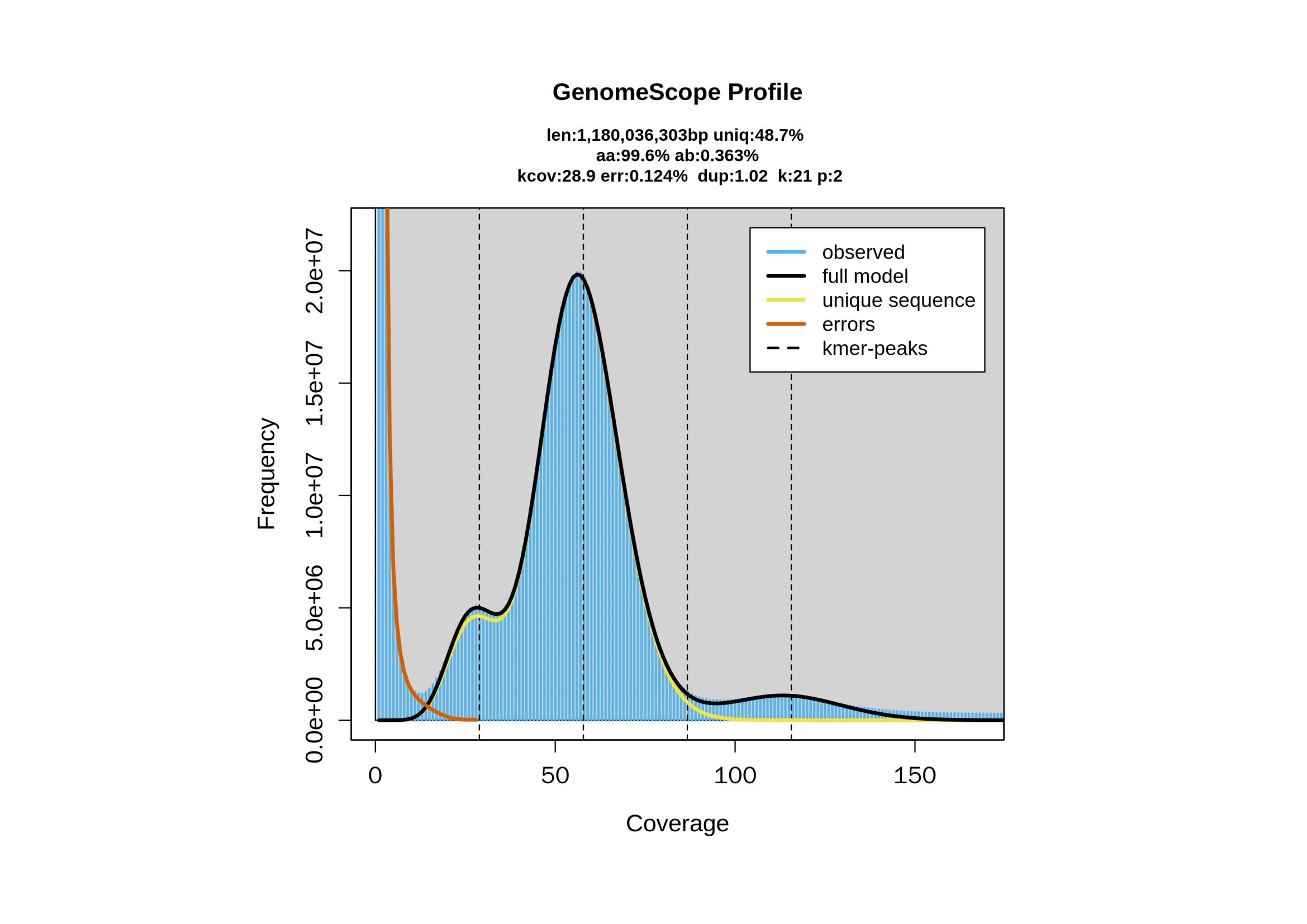

Supplement: Supplementary Figure 1 — Graph of the k-mer distribution (K = 21) generated using GenomeScope2.0. The big peak at the coverage of ∼60 in the graph is the homozygous portion of the genome, which accounts for the strands of the DNA having identical 21-mers. The smaller shoulder to the left of the peak corresponds to the heterozygous portion of the genome, which accounts for the strands of the DNA having different 21-mers. If the genome is highly heterozygous, the height of the shoulder peak would be closer to that of the homozygous peak. [file Image_1.JPEG]

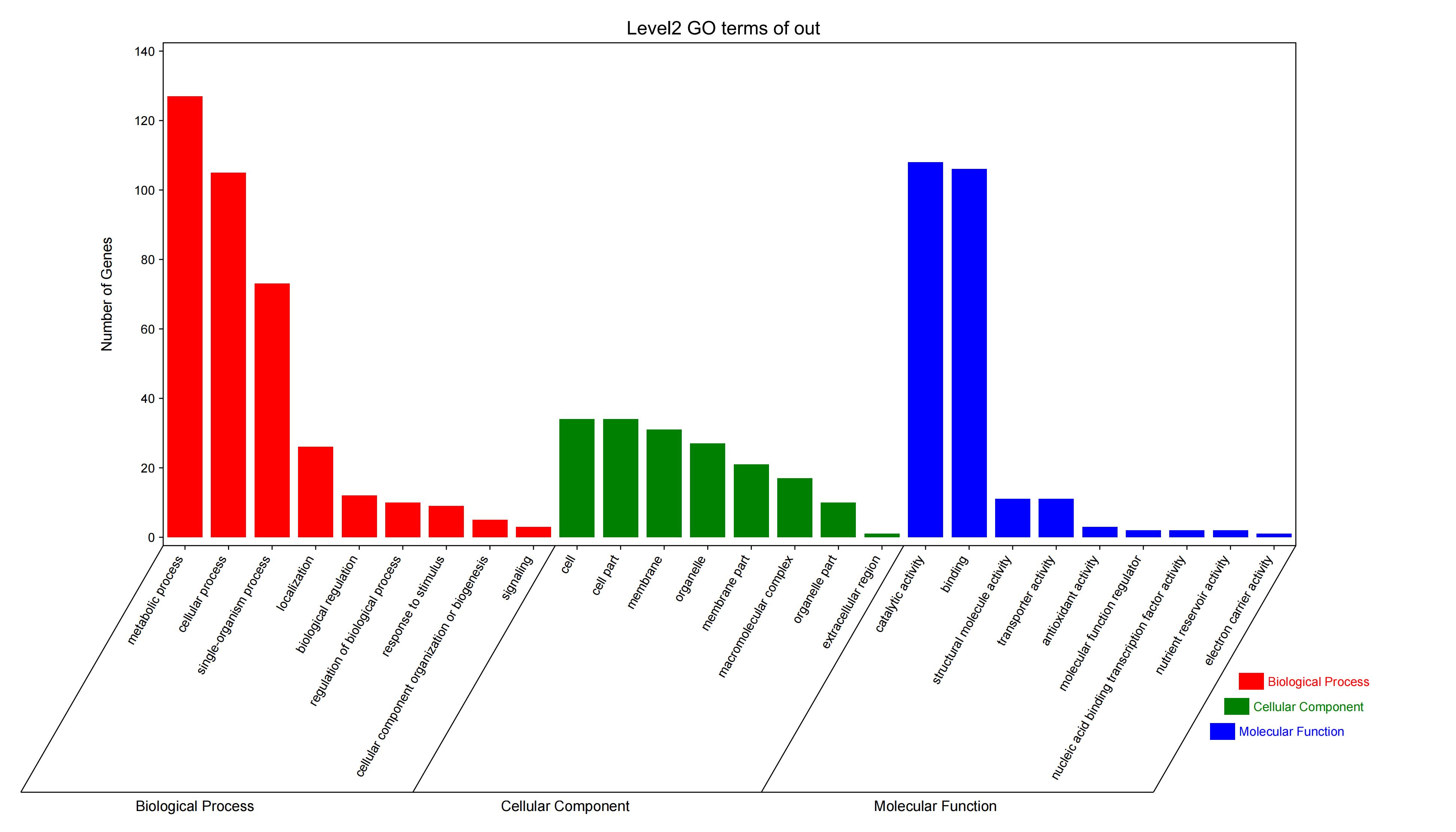

Supplement: Supplementary Figure 2 — GO terms (level 2) distribution of “Arbequina” unique genes. [file Image_2.JPEG]

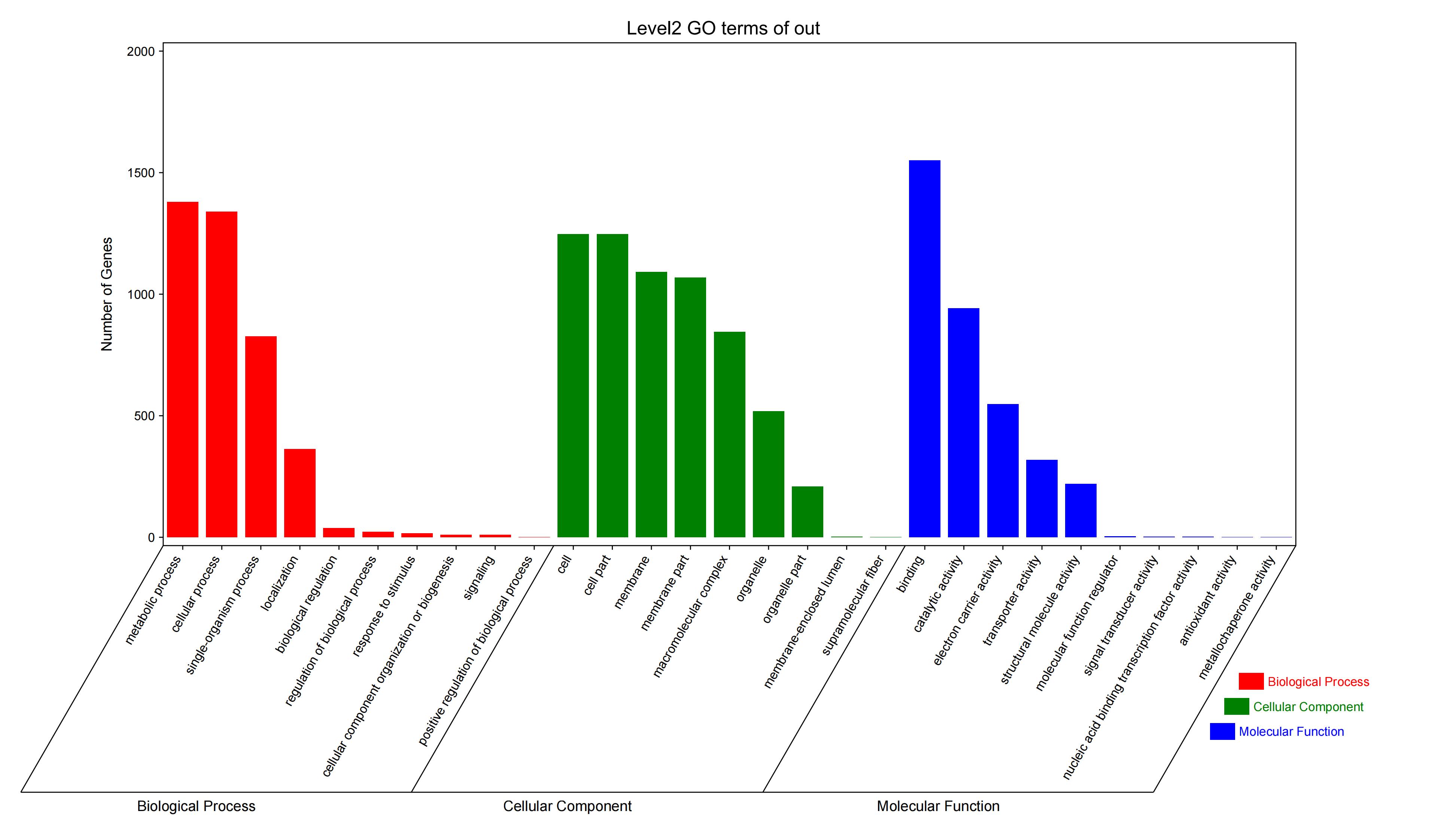

Supplement: Supplementary Figure 3 — GO terms (level 2) distribution of subsp. cuspidata unique genes. [file Image_3.JPEG]

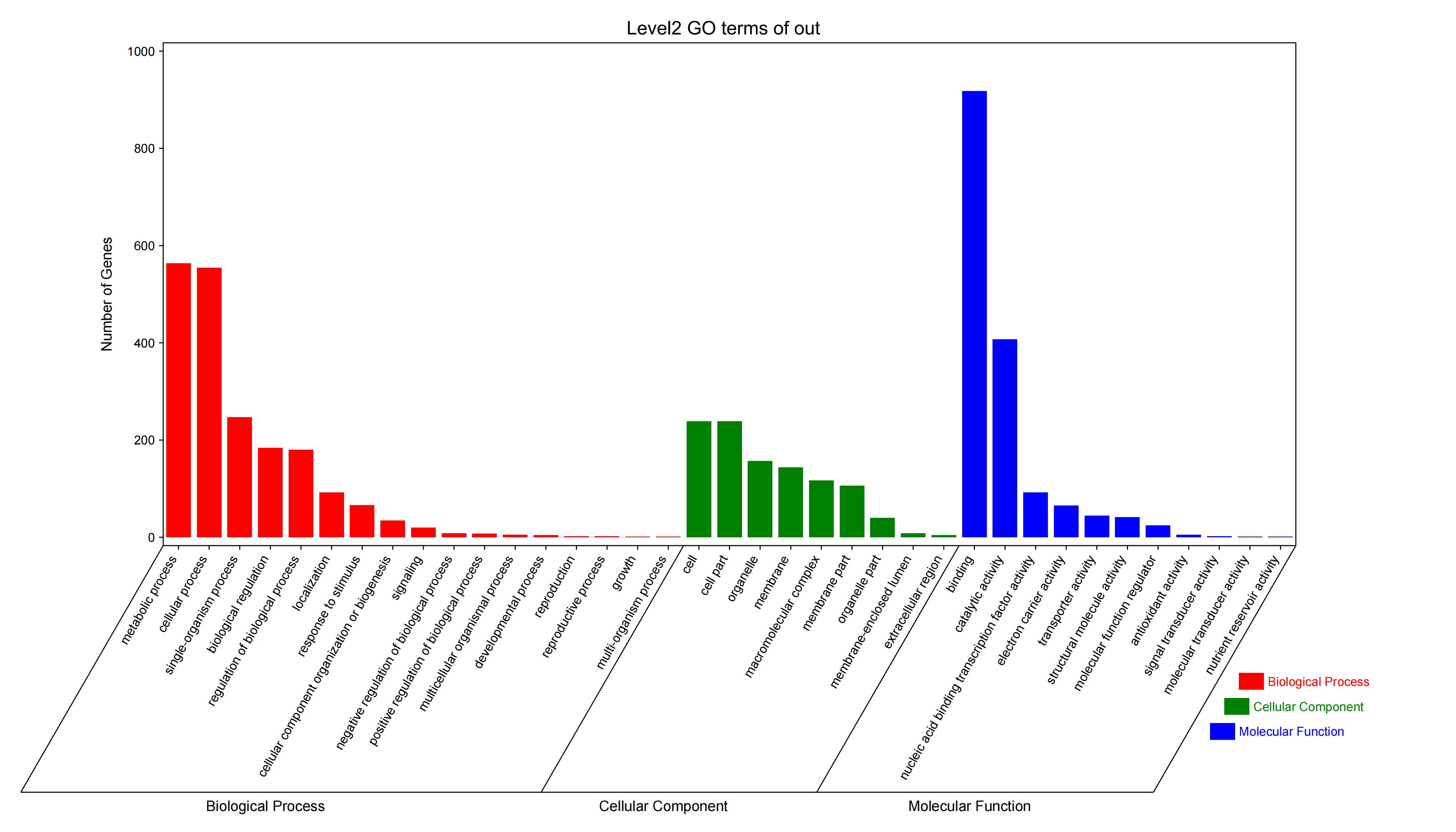

Supplement: Supplementary Figure 4 — GO terms (level 2) distribution of subsp. cuspidata positive selection genes. [file Image_4.JPEG]

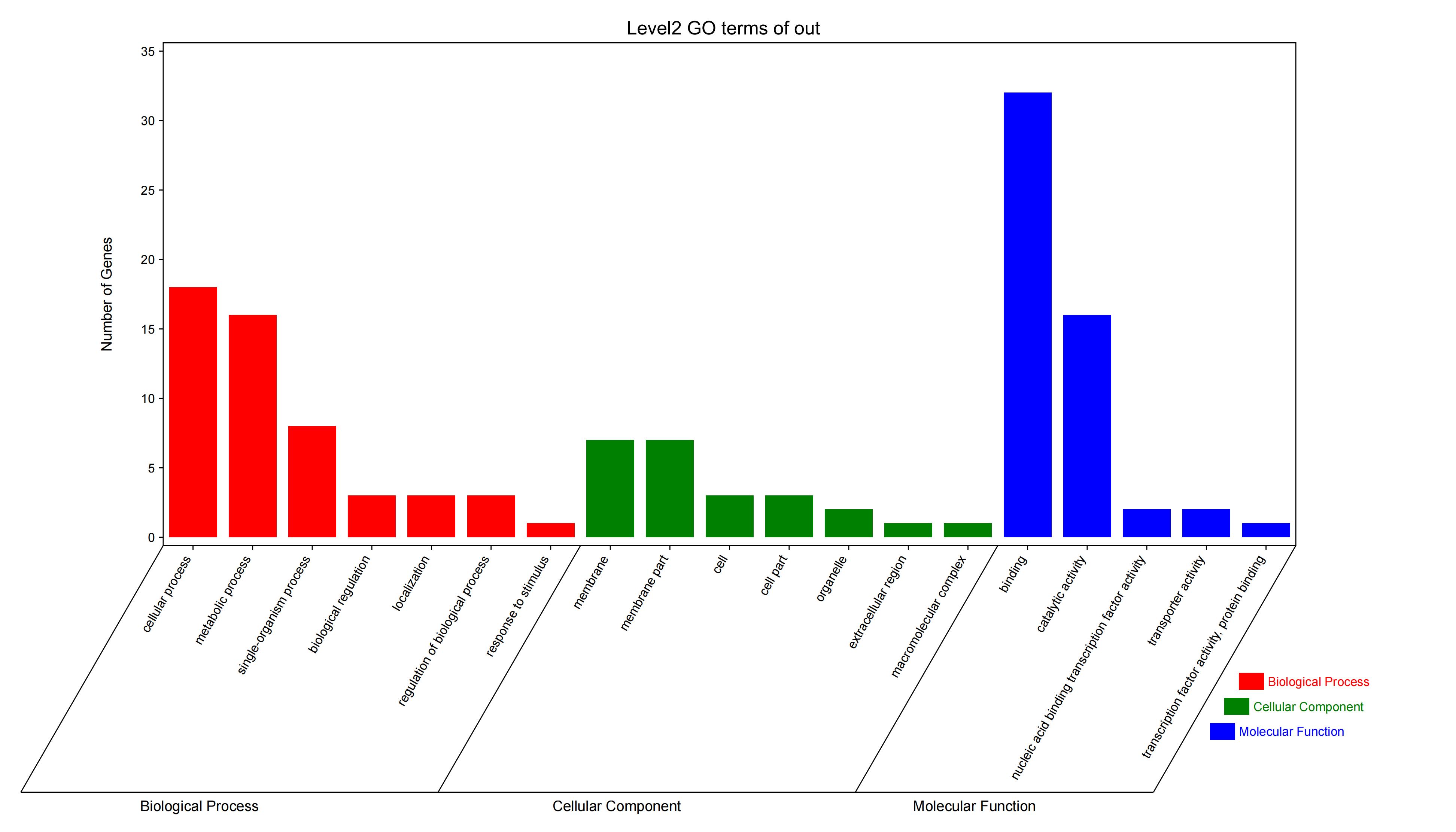

Supplement: Supplementary Figure 5 — GO terms (level 2) distribution of the 49 shared DEGs between “Arbequina” and “Arbosana”. [file Image_5.JPEG]
