# Supplementary material for: High-Quality Genome Assembly of Olea europaea subsp. cuspidata Provides Insights Into Its Resistance to Fungal Diseases in the Summer Rain Belt in East Asia
Source: Front Plant Sci. 2022 May 17;13:879822. doi: 10.3389/fpls.2022.879822 (PMC9152427; doi:10.3389/fpls.2022.879822)
Supplement: Supplementary file 6 [file Table_1.docx]

| Transcripts | Reads count | N50 (kb) | Mapping rate |
| --- | --- | --- | --- |
| leaf | 190,302 | 1.67 | 97.31% |
| root | 189,695 | 1.45 | 96.35% |
| stem | 213,361 | 1.62 | 97.43% |
| fruit | 286,357 | 1.42 | 95% |
| average |  |  | 96.52% |

**Table S1. Statistics of the transcripts of subsp. *cuspidata.***
