# Supplementary material for: High-Quality Genome Assembly of Olea europaea subsp. cuspidata Provides Insights Into Its Resistance to Fungal Diseases in the Summer Rain Belt in East Asia
Source: Front Plant Sci. 2022 May 17;13:879822. doi: 10.3389/fpls.2022.879822 (PMC9152427; doi:10.3389/fpls.2022.879822)
Supplement: Supplementary file 10 [file Table_5.docx]

**Table S5. Statistics of the fungal alignment for** **‘*Arbosana*’.**

| **Repeat** | **Query id** | **Subject id** | **Fungal species** | **Identity** | **E-value** |
| --- | --- | --- | --- | --- | --- |
| **First** | TRINITY_DN824_c0_g1_i1 | OWB81334.1 | *Candida boidinii* | 53.922 | 1.62e-07 |
|  | TRINITY_DN5439_c0_g1_i1 | XP_023460709.1 | *Cercospora beticola* | 58.273 | 2.33e-09 |
|  | TRINITY_DN1020_c0_g1_i1 | PKS02622.1 | *Cercospora zeina* | 51.852 | 5.33e-11 |
|  | TRINITY_DN5950_c0_g1_i1 | XP_018699891.1 | *Cordyceps* *fumosorosea* ARSeF 2679 | 38.288 | 4.86e-17 |
|  | TRINITY_DN1372_c0_g1_i1 | VUC24296.1 | *Clonostachys rosea* | 30.068 | 2.73e-06 |
| **Second** | TRINITY_DN3312_c0_g1_i1 | eJD40533.1 | *Auricularia subglabra* TFB-10046 SS5 | 42.929 | 1.15e-09 |
|  | TRINITY_DN141_c0_g1_i1 | XP_023460709.1 | *Cercospora beticola* | 53.75 | 4.65e-13 |
|  | TRINITY_DN2086_c0_g1_i1 | GJJ77523.1 | *entomortierella parvispora* | 39.159 | 6.05e-15 |
|  | TRINITY_DN4544_c0_g1_i1 | RDW72111.1 | *Coleophoma crateriformis* | 28.251 | 3.38e-10 |
|  | TRINITY_DN1265_c0_g1_i1 | VUC24296.1 | *Clonostachys rosea* | 29.443 | 3.98e-09 |
|  | TRINITY_DN1265_c0_g1_i2 | KAH7017169.1 | *Ilyonectria destructans* | 30.278 | 5.45e-09 |
| **Third** | TRINITY_DN4697_c0_g1_i1 | KAF7192872.1 | *Pseudocercospora fuligena* | 44.444 | 8.48e-06 |
|  | TRINITY_DN814_c0_g1_i1 | XP_023460709.1 | *Cercospora beticola* | 56.911 | 2.99e-07 |
|  | TRINITY_DN954_c0_g1_i1 | KAH7134006.1 | *Dactylonectria macrodidyma* | 31.304 | 8.02e-08 |
|  | TRINITY_DN1910_c0_g1_i1 | GJJ77523.1 | *entomortierella parvispora* | 39.568 | 5.35e-09 |
|  | TRINITY_DN4760_c0_g2_i1 | KAH6995966.1 | *Ilyonectria* sp. MPI-CAGe-AT-0026 | 33.984 | 4.56e-09 |
|  | TRINITY_DN3726_c0_g1_i1 | KAG2127318.1 | *Suillus cothurnatus* | 42.286 | 1.96e-14 |
