# Supplementary material for: High-Quality Genome Assembly of Olea europaea subsp. cuspidata Provides Insights Into Its Resistance to Fungal Diseases in the Summer Rain Belt in East Asia
Source: Front Plant Sci. 2022 May 17;13:879822. doi: 10.3389/fpls.2022.879822 (PMC9152427; doi:10.3389/fpls.2022.879822)
Supplement: Supplementary file 11 [file Table_6.docx]

**Table S6. Statistics of the fungal alignment for ‘*Arbequina*’.**

| **Repeat** | **Query id** | **Subject id** | **Fungal species** | **Identity** | **E-value** |
| --- | --- | --- | --- | --- | --- |
| **First** | TRINITY_DN1466_c1_g4_i1 | XP_044656707.1 | *Cercospora kikuchii* | 49.673 | 5.74e-07 |
|  | TRINITY_DN6169_c0_g1_i1 | GJJ77523.1 | *entomortierella parvispora* | 34.892 | 1.84e-08 |
|  | TRINITY_DN919_c1_g1_i1 | GJJ77523.1 | *entomortierella parvispora* | 38.021 | 1.11e-13 |
|  | TRINITY_DN4561_c0_g1_i1 | KAF8509459.1 | *Hysterangium stoloniferum* | 33.333 | 8.66e-07 |
|  | TRINITY_DN4532_c0_g1_i1 | GJJ77523.1 | *entomortierella parvispora* | 41.667 | 8.60e-11 |
|  | TRINITY_DN4432_c0_g1_i1 | XP_044656707.1 | *Cercospora kikuchii* | 40.435 | 1.12e-13 |
|  | TRINITY_DN3522_c0_g1_i1 | OWB81334.1 | *Candida boidini* | 48.507 | 1.19e-07 |
|  | TRINITY_DN1829_c0_g2_i1 | GJJ77523.1 | *entomortierella parvispora* | 39.489 | 6.53e-06 |
| **Second** | TRINITY_DN838_c0_g1_i1 | KAF7192872.1 | *Pseudocercospora fuligena* | 36.494 | 1.49e-17 |
|  | TRINITY_DN838_c0_g2_i1 | KAF7192872.1 | *Pseudocercospora fuligena* | 35.887 | 1.75e-09 |
|  | TRINITY_DN4316_c0_g1_i1 | XP_044656707.1 | *Cercospora kikuchii* | 62.963 | 5.84e-18 |
|  | TRINITY_DN495_c0_g1_i1 | KAG2127318.1 | *Suillus cothurnatus* | 41.477 | 3.88e-15 |
|  | TRINITY_DN299_c0_g1_i1 | XP_044656707.1 | *Cercospora kikuchii* | 59.259 | 8.49e-10 |
|  | TRINITY_DN4575_c0_g1_i1 | KAH6629021.1 | *Boeremia exigua* | 61.364 | 9.35e-11 |
|  | TRINITY_DN5732_c0_g1_i1 | XP_023460709.1 | *Cercospora beticola* | 49.275 | 4.56e-13 |
|  | TRINITY_DN7113_c0_g1_i1 | GJJ77523.1 | *entomortierella parvispora* | 34.47 | 6.77e-09 |
|  | TRINITY_DN532_c0_g1_i1 | GJJ77523.1 | *entomortierella parvispora* | 35.678 | 1.28e-09 |
|  | TRINITY_DN1354_c0_g1_i1 | KAF5095562.1 | *Geotrichum candidum* | 38.012 | 4.56e-08 |
|  | TRINITY_DN1354_c0_g1_i2 | KAF5105306.1 | *Geotrichum candidum* | 37.778 | 1.61e-06 |
| **Third** | TRINITY_DN2255_c0_g1_i1 | KAF7192872.1 | *Pseudocercospora fuligena* | 41.784 | 3.51e-06 |
|  | TRINITY_DN1_c0_g1_i1 | RDW59157.1 | *Coleophoma crateriformis* | 52.143 | 1.31e-10 |
|  | TRINITY_DN4026_c0_g1_i1 | XP_044656707.1 | *Cercospora kikuchii* | 55 | 1.23e-09 |
|  | TRINITY_DN7103_c0_g1_i1 | GJJ77523.1 | *entomortierella parvispora* | 38.506 | 1.08e-11 |
|  | TRINITY_DN2832_c0_g1_i1 | OWB81334.1 | *Candida boidini* | 56.701 | 4.40e-08 |
